# Clinical performance of a smartphone-based low vision aid

**DOI:** 10.1038/s41598-022-14489-z

**Published:** 2022-06-24

**Authors:** Joon Hyung Yeo, Seon Ha Bae, Seung Hyeun Lee, Kyoung Woo Kim, Nam Ju Moon

**Affiliations:** 1 Department of Ophthalmology, Chung-Ang University Gwangmyeong Hospital, Chung-Ang University College of Medicine, Gwangmyeong, Republic of Korea; 2 Department of Ophthalmology, Chung-Ang University Hospital, Chung-Ang University College of Medicine, Seoul, Republic of Korea

**Keywords:** Eye diseases, Eye manifestations

## Abstract

Real-time digital image processing to optimally enhance low vision is now realizable with recent advances in personal computers. This study aimed to evaluate the efficacy of a wearable smartphone-based low vision aid (LVA) with customizable vision enhancement in patients with visual impairment. We recruited 35 subjects with visual impairment and who were literate and cognitively capable. The subjects completed a training session and were provided a smartphone-based LVA for a 4-week use. Visual functions including binocular best-corrected distance, intermediate, and near visual acuities; reading performance (reading speed and accuracy); and facial recognition performance were measured at baseline and after 4-weeks use. All subjects also completed the Low Vision Quality of Life (LVQOL) Questionnaire. Thirty-four subjects (mean age, 43.82 ± 15.06 years) completed the study. Significant improvements in binocular best-corrected distance, intermediate, and near visual acuities were observed after smartphone-based LVA use (all *p* < 0.001). Reading accuracy and facial recognition performance also improved significantly (*p* = 0.009 and *p* < 0.001, respectively), but reading speed did not. LVQOL scores significantly improved after 4 weeks of use in subjects aged < 40 years (*p* = 0.024), but not in subjects aged ≥ 40 years (*p* = 0.653). Ocular and non-ocular adverse events were infrequent and resolved when the device was removed. The smartphone-based LVA with customizable vision enhancement could provide clinically significant improvements in the visual function of patients with visual impairment and was generally well tolerated. This study suggests that the smartphone-based LVA would be beneficial for visual rehabilitation.

## Introduction

Low vision is a major disability that has a profound impact on both personal and professional aspects. The associated vision impairment results in severe difficulty with or inability to perform daily living activities^1,2^. Consequently, low vision can have a significant impact on the patients’ participation in activities of interest, independence, social interactions, quality of life, and ultimately, emotional health^3–6^. Patients who fail medical or surgical treatment are provided low vision aids (LVAs) to maximize residual vision and improve the patients’ quality of life^7^.

Common LVAs include optical (e.g., hand magnifiers, stand magnifiers, and telescopes), non-optical (e.g., reading stand, typoscopes, and sunglasses), and digital (e.g., closed-circuit television [CCTV] and portable digital magnifiers) devices, which are widely available in handheld and desk-mounted formats. Advances in display and image processing techniques allow for various electronic LVAs to aid patients with visual impairment. Especially, head-mounted display (HMD) systems equipped with forward-looking video cameras offer the user a hands-free format and have been used in low vision rehabilitation for more than two decades^8–10^. However, limitations, including a stigmatizing design, device weight, limited field of view, resolution, battery life, and high price, have led to a low demand on the market.

With the recent advent of personal computers (e.g., smartphones), real-time digital image processing to optimally enhance low vision is now realizable. These techniques include virtual vision, in which a display replaces natural vision, and augmented vision, in which a display adds to the natural vision. These technological advancements in smartphone computerization and virtual reality (VR) and augmented reality (AR) have led to the development of a novel and more advanced LVA: smartphone-based LVA. There are two commercially available smartphone-based head-mounted LVAs: IrisVision (irisvision.com) and SightPlus (givevision.net). Although the two devices have a common concept of a smartphone (serving as a camera, image processor, and display) inserted into a VR headset serving as the viewing system, each device has a different digital image enhancement strategy. However, none of the two LVAs is designed to provide customized image enhancement that is optimal for individual patients.

This study aimed to assess the effects of a smartphone-based LVA with customizable vision enhancement on visual performance (as assessed by visual acuity, reading performance, and face recognition performance) and quality of life in patients with visual impairment.

## Results

Of the 38 individuals who were screened for participation, 35 individuals met the inclusion criteria and were enrolled in the study. One subject was lost to follow-up and did not complete the study. Therefore, 34 subjects were included in the analyses. The subject characteristics are summarized in Table 1. Briefly, the mean subject age was 43.82 ± 15.06 years (range: 19–76 years), and 19 subjects (55.88%) were female. The causes of visual impairment differed widely; seven subjects had optic atrophy; five subjects, inherited macular diseases; four subjects, congenital cataract; four subjects, retinitis pigmentosa; four subjects, age-related macular disease; two subjects, albinism; and two subjects, glaucoma. Congenital nystagmus, congenital nanopthalmos, multiple sclerosis, Leber hereditary optic neuropathy, retinal detachment, and proliferative diabetic retinopathy were found in one subject each. At the time of enrollment, 22 subjects (64.7%) had experience with other LVAs. Of the 22 subjects, 18 had a single LVA and 4 had two or more LVAs; 9 subjects had portable digital magnifiers; 7 subjects, CCTV; 6 subjects, magnifiers for near vision tasks; and 3 subjects, telescopes for distant vision tasks. One subject used a text-to-speech device.

**Table 1:** Clinicodemographic characteristics of study subjects.

Characteristics	n = 34
**Age, years, mean (SD)**	43.82 (15.06)
Less than 20 years, *n (%)*	2 (5.88)
20–29 years, *n (%)*	6 (17.65)
30–39 years, *n (%)*	4 (11.76)
40–49 years, *n (%)*	10 (29.41)
50–59 years, *n (%)*	7 (20.59)
60–69 years, *n (%)*	3 (8.82)
70–79 years, *n (%)*	2 (5.88)
**Females, n (%)**	19 (55.88)
**Cause of visual impairment, n (%)**	
Optic atrophy	7 (20.59)
Inherited macular disease	5 (14.71)
Retinitis pigmentosa	4 (11.76)
Age-related macular disease	4 (11.76)
Congenital cataract	4 (11.76)
Albinism	2 (5.88)
Glaucoma	2 (5.88)
Proliferative diabetic retinopathy	1 (2.94)
Congenital nystagmus	1 (2.94)
Congenital nanophthalmos	1 (2.94)
Retinal detachment	1 (2.94)
Multiple sclerosis	1 (2.94)
Leber hereditary optic neuropathy	1 (2.94)

*SD* standard deviation.

### Visual acuity

The mean logarithm of the minimum angle of resolution (logMAR) binocular best-corrected distance, intermediate, and near visual acuities (BCDVA, BCIVA, and BCNVA, respectively) at baseline (Visit 1) were 1.07 ± 0.24, 1.15 ± 0.34, and 1.11 ± 0.39, respectively. All these parameters were significantly improved after the initial training session (Visit 2, all *p* < 0.001). After 4 weeks of use, the logMAR BCDVA, BCIVA, and BCNVA were significantly improved to 0.09 ± 0.15, 0.33 ± 0.09, and 0.18 ± 0.22, respectively (all *p* < 0.001). Post hoc analysis revealed significant improvements in BCDVA and BCNVA, but not in BCIVA, between Visit 2 and Visit 3 (Table 2).

**Table 2:** Visual function measured at each visit.

	Visit 1 (baseline)	Visit 2 (after training session)	Visit 3 (after 4 weeks of home use)	*p* value*	*p* value^†^
**Binocular BCVA, logMAR**					
Distance	1.07 (0.24)	0.16 (0.20)	0.09 (0.15)	< 0.001	0.001
Intermediate	1.15 (0.34)	0.35 (0.15)	0.33 (0.09)	< 0.001	0.188
Near	1.11 (0.39)	0.30 (0.21)	0.21 (0.26)	< 0.001	0.001
**Reading performance**					
Reading speed, lpm	143.19 (96.13)	151.61 (98.65)	147.44 (96.42)	0.857	0.861
Reading accuracy, %	86.94(26.77)	95.95 (14.58)	97.17 (9.06)	0.009	0.682
**Face recognition performance, score**					
	49.86 (13.68)	–	55.99 (14.78)	< 0.001	–
**LVQOL questionnaire, score**					
Total	64.06 (17.47)	–	64.59 (17.84)	0.803	–
Age < 40 years	69.33 (9.55)	–	78.08 (9.88)	0.024	–
Age ≥ 40 years	60.09 (17.35)	–	59.86 (16.37)	0.653	–

Values are presented as the mean (standard deviation).

*BCVA* best-corrected visual acuity, *logMAR* logarithm of minimum angle of resolution, *lpm* letter per minute, *LVQOL* low vision quality of life.

*Statistical comparison between Visit 1 and Visit 3.

^†^Statistical comparison between Visit 2 and Visit 3.

### Reading performance and face recognition performance

As shown in Table 2, reading speed did not significantly change with smartphone-based LVA use (143.19 ± 96.13 letters per minute [lpm] vs. 147.44 ± 96.42 lpm, *p* = 0.857). However, reading accuracy significantly improved (86.94 ± 26.77% vs. 97.17 ± 9.06%, *p* = 0.009). In addition, the smartphone-based LVA significantly improved face recognition performance (*p* < 0.001). There was no further improvement after 4 weeks of use.

### Low vision quality-of-life questionnaire and user-feedback

Although Low Vision Quality of Life (LVQOL) scores did not show a statistically significant change after 4 weeks of smartphone-based LVA use, subgroup analysis revealed that LVQOL scores significantly improved from 69.33 ± 9.55 at baseline to 78.08 ± 9.88 after 4 weeks of use (*p* = 0.024) in subjects aged < 40 years, but not in subjects aged ≥ 40 years (Table 2). We used stepwise multivariate regression analysis to identify the predictor variables related to the improvement of the LVQOL score. The results of univariate and multivariate analyses are shown in Table 3. In the multivariate analysis, younger age (*p* = 0.002), better baseline near visual acuity (*p* = 0.011), and previous use of LVAs (*p* = 0.049) were associated with a greater improvement in LVQOL score, whereas average time-of-use per use, number of use per day, and total time-of-use per day were not. In addition, LVQOL score improvement was not related to the patients’ baseline LVQOL score or BCDVA, BCIVA, and BCNVA with smartphone-based LVA.

**Table 3:** Univariate and multivariate linear regression analyses of the association between clinicodemographic factors and improvement of LVQOL score.

	Univariate			Multivariate		
	*B*^a^	B*	*p* value	*B*^a^	*B*^†^	*p* value
Age, years	− 0.566	− 0.478	0.001	− 0.558	− 0.472	0.002
Sex	− 1.095	− 0.031	0.862			
Previous use of LVAs	7.137	0.202	0.041	10.925	0.309	0.049
Baseline BCDVA, logMAR	− 14.106	− 0.191	0.279			
Baseline BCIVA, logMAR	− 10.314	− 0.198	0.262			
Baseline BCNVA, logMAR	− 14.783	− 0.324	0.006	− 17.825	− 0.390	0.011
BCDVA with smartphone-based LVA, logMAR	− 0.117	− 0.783	0.440			
BCIVA with smartphone-based LVA, logMAR	0.105	0.698	0.491			
BCNVA with smartphone-based LVA, logMAR	− 0.040	− 0.245	0.808			
Baseline face recognition performance, score	0.489	0.375	0.029	0.089	0.093	0.619
Number of smartphone-based LVA use per day	0.798	0.038	0.829			
Average time-of-use per use, minutes	− 0.022	− 0.031	0.860			
Average total time-of-use per day, minutes	0.034	0.084	0.686			

*LVQOL* low vision quality of life, *LVA* low vision aid, *BCDVA* best-corrected distance visual acuity, *logMAR* logarithm of minimum angle of resolution, *BCIVA* best-corrected intermediate visual acuity, *BCNVA* best-corrected near visual acuity.

*Unstandardized β coefficient.

^†^Standardized β coefficient.

User feedback showed that a smartphone-based LVA was be effective in performing daily activities that are often difficult for patients with visual impairment, including face recognition, television or movie watching, near reading (newspaper, book, menu, or labels on medicine bottles), computer or smartphone watching, distance reading (distant signs or a clock), and letter writing (Table 4). Additionally, 32 out of 34 subjects (94.12%) showed a willingness to purchase at the end of the study and 21 of 22 subjects (95.45%) who were already using a LVA at baseline preferred using the smartphone-based LVA over their prior LVAs.

**Table 4:** Summary of user feedback.

Questions	
**How many times did you use Relumino per day?**	n = 34
≤ 1 time/day, n (%)	13 (38.24)
≤ 2 time/day, n (%)	11 (32.35)
> 2 time/day, n (%)	10 (29.41)
**How long had you been using Relumino on average at once?**	n = 34
Less than 30 min, n (%)	13 (38.24)
30–60 min, n (%)	13 (38.24)
More than 60 min, n (%)	8 (23.53)
Which activities did you think Relumino would be effective?*	
Television or movie watching	20
Near reading	17
Smartphone or computer watching	13
Distance reading	7
Face recognition	3
Letter writing	3
**I prefer the Relumino to previous low vision aid**	n = 22^†^
Yes, n (%)	21 (95.45)
No, n (%)	1 (4.55)
**If available, I would buy the Relumino**	n = 34
Yes, n (%)	32 (94.12)
No, n (%)	2 (5.88)

*Multiple answers are possible.

^†^Applied to subjects who had an experience with low vision aid.

### Safety and corneal surface index

The smartphone-based LVA was generally well tolerated and no serious adverse events occurred in this study. The overall ocular and non-ocular adverse events are listed in Table 5. The most common ocular adverse events were eye strain and dryness (6 subjects, 17.64%). All the dry eye cases were mild, treated with artificial tears, and resolved with no sequelae. With regard to non-ocular adverse events, 5 subjects (14.71%) reported dizziness, which resolved as soon as the device was removed. Table 6 shows the corneal surface index of the subjects. Briefly, no significant changes were observed in the TBUT, CFS, and Schirmer test scores.

**Table 5:** Adverse events.

Adverse events	n (%)
**Ocular**	7 (20.59)
Eye strain	3 (8.82)
Eye dryness	3 (8.82)
Glare	1 (2.94)
**Non-ocular**	6 (17.65)
Dizziness	5 (14.71)
Device site pain	1 (2.94)

**Table 6:** Corneal surface indices.

	Visit 1	Visit 3	*p* value
Tear film break-up time, sec	5.06 (1.43)	5.61 (1.71)	0.402
Corneal fluorescein staining, score	0.76 (1.30)	0.66 (1.77)	0.698
Schirmer I test, mm	15.07 (10.58)	14.84 (10.75)	0.897

Values are presented as the mean (standard deviation).

## Discussion

The smartphone-based LVA examined in the current study was generally well tolerated and provided clinically significant improvements in visual function in patients with visual impairment. Overall, wearing the smartphone-based LVA instantly improved the subjects’ distance, intermediate, and near visual acuities; reading accuracy; and facial recognition performance. After 4 weeks of use, subjects aged younger than 40 years were more likely to be satisfied with the smartphone-based LVA.

Ehrlich et al*.* reviewed HMDs for patients with visual impairment and highlighted the advantages of such technology over conventional desk-mounted or handheld sight aids^11^. Recent advances in technology have led to the availability of smaller, lighter, and more versatile electronic head-mounted LVA^12,13^. However, to date, only one study has reported the clinical benefit of a smartphone-based head-mounted LVA in clinical settings (SightPlus®)^14^. SightPlus included five pre-set image enhancement modes, and the study found improvements in distant and near visual acuities and contrast sensitivity in 60 patients with visual impairment^14^.

Similarly, the present study also found improvements in visual acuities. We reported a mean change in BCDVA of 0.98 ± 0.20 logMAR, which was comparable to the 0.63 ± 0.34 logMAR with SightPlus. On this basis, the non-inferiority of Relumino to SightPlus was demonstrated. However, 47% of the subjects in the previous study indicated that they would use SightPlus, while 94% of the subjects in the current study indicated that they would use Relumino. Although there was no direct comparison between the two devices, we thought that a possible reason for the higher preference for Relumino was the ability to provide a vision enhancement format customized to each patient. Indeed, Relumino allows patients to make user-specific vision enhancement formats with a combination of multiple functions such as color filtering, edge/text enhancement, and a variable levels of magnification, while SightPlus has a pre-set image enhancement mode. An advantage of HMDs may be that a wide range of magnification provides a benefit for resolution tasks at far, intermediate, and near distances. Considering that no patient used HMD at the time of enrollment, it is also possible that the benefits of HMD itself may have resulted in a high preference for Relumino in this study.

The current study found that a smartphone-based LVA significantly improved the face recognition performance, while a prior study demonstrated that magnification itself had a limited effect on emotion detection in patients with age-related macular degeneration^15^. This finding might result from a visual enhancement strategy using magnification combined with contrast enhancement. Therefore, we believed that smartphone-based LVAs customized to each patient would become more important in the personal and vocational rehabilitation of the visually impaired.

For reading performance, the use of the smartphone-based LVA improved reading accuracy but had little effect on reading speed. This is in line with a previous study that reported a significant decrease in reading speed^14^. The insignificant effect on reading speed could be explained by the reduced field of view. Most patients with visual impairment, including our study subjects, require magnification for near-distance visual tasks, which results in a reduced field of view. Unfortunately, within this narrow field of view, patients with visual impairment are likely to lose their place on a page of text. Virgili et al*.* also found that reading speed may be higher in stand-mounted electronic CCTV than in head-mounted devices^16^. Moreover, after capturing images through the smartphone camera, the smartphone-based LVA process images and display the modified images. The image processing results in an approximately 50 ms of delay, making word tracking difficult. Despite the reduced reading speed in our study, a significant number of subjects reported that the smartphone-based LVA was effective in near reading, perhaps because of the increased working distance or comfort of reading (none of which we assessed).

Although subjects showed improvements in visual function with the use of smartphone-based LVA, no favorable outcome in the LVQOL score was reported. Considering that the majority of electronic LVA users are young and highly motivated^8,17^, we hypothesized that young users were more likely to show LVQOL improvement than elderly users. As a result, our univariate and multivariate analyses showed that young age, better baseline near visual acuity, and previous use of other LVAs were predictors for improvement of quality of life. Additional analysis regarding the LVQOL scores showed that the scores in subjects aged < 40 years significantly improved after 4 weeks of use, but not in subjects aged ≥ 40 years. This finding is in line with previous studies that reported that younger age is a predictor of better compliance to LVAs^10,18^. However, despite the difference in the improvement of LVQOL in the two age groups, there was no difference in the improvement of visual acuity according to age. In addition, it should be noted that all subjects had no prior experience with HMDs and only became familiar with them during the study. Further investigation into the practice effects and structured training would help determine whether improvement of LVQOL in elderly patients is possible.

With respect to tolerability, the smartphone-based LVA was generally well tolerated. The most common reported side effects were eye strain and dryness, which have been reported as side effects of wearable devices^12,19^. However, there was no aggravation in corneal surface index after the use of the smartphone-based LVA. Thus, we believe that moderate use of a smartphone-based LVA for 1–2 h per day is less likely to cause dry eye. Future studies should collect additional data about the safety profile after long-term device use. Another drawback is the size and bulkiness of the devices. They tend to protrude in front of the face and can be uncomfortable for long-time use. Moreover, the HMD systems made subjects anxious about using the device outside of their homes. This is in agreement with previous studies that showed patients with visual impairment fear having stereotypes, and LVA use in public can worsen this fear because they are symbolic of vision loss^20^.

The current study had some limitations. First, our study did not compare device performance with that of other LVAs. However, of the 22 subjects who were already using other LVAs at the time of enrollment, user feedback showed that almost all of them (21/22 subjects) preferred using the smartphone-based LVA over their prior LVAs. Future studies should include direct comparisons between the smartphone-based LVA and other LVAs. Second, clinical heterogeneity regarding the difference in patient age, cause of vision loss, and visual field deficit may affect the clinical benefits of smartphone-based LVAs. Especially, as a relatively small number of elderly patients were included in this study, our findings should be confirmed in a broad range of age groups for further generalization. Third, we allowed subjects to freely choose the image enhancement mode that they found most useful for a given task, but the data about enhancement-mode preferences for each task were not documented. In addition, we evaluated visual function in the clinical settings. Given that real-world images or scenes differ from those of clinical settings and have a broader range of colors and contrasts, future studies that represent different types of real-life scenarios are required. Fourth, although regular phone call follow-up was made weekly, it is not necessarily an adequate substitute for a training program administered by a qualified instructor. It would be better to arrange a home visit to check how participants are using the smartphone-based LVA and to conduct a home exercise program to improve patients’ acceptability and enhance visual performance. Lastly, to support the clinical benefit of smartphone-based LVA and to examine the predictive factors of benefit (e.g., specific disease condition, age, and baseline visual acuity cut-offs), well-designed large-scale trials with longer study periods are required.

In conclusion, a smartphone-based LVA with customizable vision enhancement has a favorable effect on the visual function of patients with visual impairment and is well-tolerated. We hope that future technological advances will further improve the convenience of the smartphone-based LVA (e.g., faster processors, smaller components, lighter materials), its performance, and acceptability so that it can be more widely used in visual rehabilitation.

## Methods

This prospective, single-arm study was approved by the Institutional Review Board of Chung-Ang University Hospital (IRB No. 1620-003-262) and adhered to the tenets of the Declaration of Helsinki. Written informed consent was obtained from all participants after explanation of the nature and possible consequences of study participation.

### Study subjects

All subjects were recruited between September and November 2020 from the patient pool of the low vision clinic at Chung-Ang University Hospital. Subjects with visual impairment, defined as a best-corrected visual acuity of 20/60 to 20/400 in the better eye and had been stable for at least 6 months, due to any cause were eligible. Patients who had a cognitive impairment, could not read, or had a physical disability that made smartphone-based LVA operation difficult were excluded.

All subjects underwent baseline measurements without the device with best correction (Visit 1). Within 2 weeks of study enrollment, all subjects underwent a training session and measurements with the device (Visit 2). After 4 weeks (± 1 week) of home use, the subjects returned to the clinic and completed their final measurements (Visit 3).

### Smartphone-based low vision aid

**Figure 1:**
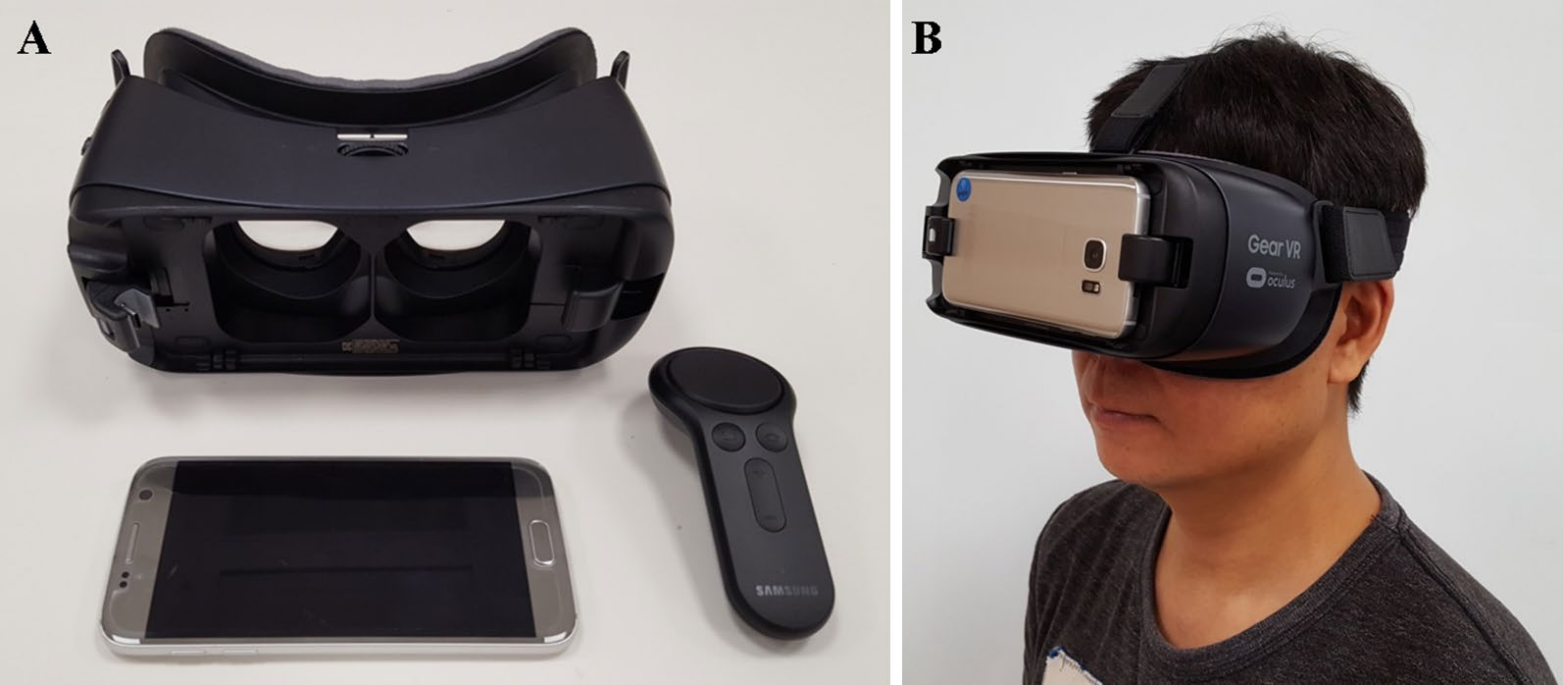
Smartphone-based low vision aid (LVA). (**A**) The smartphone-based LVA is composed of the VR head-mounted display, a smartphone, smartphone application software, and a remote control. (**B**) Photograph of a patient wearing the smartphone-based LVA. The operation remote is held in the patient’s hand.

**Figure 2:**
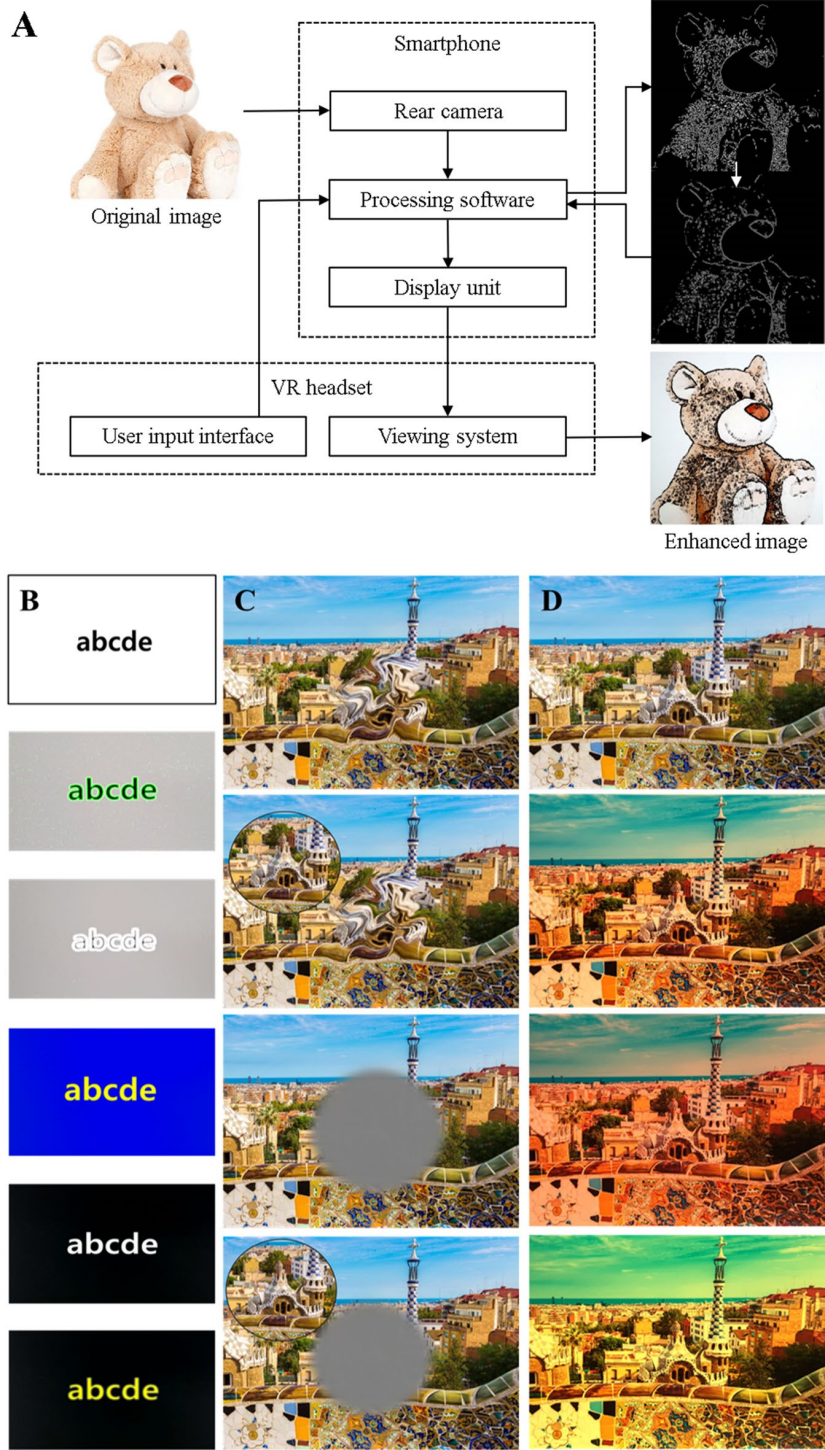
Representative images of the function of smartphone-based LVA. (**A**) For edge enhancement, the internal software of Relumino® modifies the original images obtained by the rear camera of the smartphone to enhanced images. Specifically, the processing software detects vertical and horizontal edges and converts edge images into a binary image. After noise reduction and edge magnitude adjustment, binary images are superimposed with the original images. These enhanced images are then transmitted to the binocular viewing system of the VR headset. Similarly (each column begins with the original image and is followed by these modifications), a smartphone-based LVA can also provide text enhancement (**B**, applied to text to increase visibility by controlling contrast and spatial frequency), image remapping (**C**, used to remap a distorted image or image falling on the scotoma to another location on the screen), and color filtering (**D**).

The hardware and software components of smartphone-based LVA (Relumino®) included a VR headset (Gear VR, Samsung Electronics, Suwon-si, Gyeonggi-do, Korea) that worked with an inserted smartphone (Galaxy S8, Samsung Electronics), embedded Relumino software (Samsung Electronics, https://www.samsungrelumino.com), and a handheld remote controller (Fig. 1). The device's weight was 500 g. The smartphone had a total screen resolution of 2960 × 1440 pixels (570 pixels per inch) and presented a digital image on an AMOLED screen. The headset’s lenses provided an approximately 110 degrees diagonal field of view and offered up to 10 × magnification, while the software offered up to 8 × magnification. The Relumino software processed the image projected through the rear camera of the smartphone, and then displayed modified images in the binocular viewing system of the VR headset in real-time (Fig. 2A). For edge/text enhancements, original images were modified to emphasize the object outlines for augmented image recognition. A sobel filter was used to detect vertical and horizontal edges. In addition, two image remapping algorithms allowed the user to relocate the images falling on the scotoma to a preferred retinal locus and to shrink the entire image to place in the spared retina. The Relumino software was designed to provide not only edge/text enhancement and image remapping but also linear magnification, autofocus, contrast enhancement, image freezing, and color filtering (Fig. 2B). When activated, the user can choose to use augmented vision enhancements (edge/text enhancements [user-selected color and contrast], color filtering, and contrast enhancement) or virtual vision enhancement (image remapping). Moreover, a smartphone-based LVA was programmed to be customized to the individual patient through adjustment and a combination of multiple functions (e.g. magnification with edge enhancement).

### Smartphone-based low vision aid training

At Visit 2, all subjects and their family completed a 90-min training session. Subjects were given instructions on how to use the device and to ideally adjust the settings for their viewing. Specifically, the instructor gave visual tasks which mimic real-world situations (such as reading text messages on the smartphone, watching TV or Youtube, filling application forms, watching a clock on the wall, etc.) and encouraged the subjects to try to adjust the zoom, focus, contrast, color filter, or image-freezing. As a given task was being performed, the instructor could see the image that the patient was viewing via a mirroring Bluetooth connection to a remote monitor and advise on image-processing strategies to optimize vision enhancement. The training course was administered by a single instructor (S.J.H.) with extensive experience in low vision rehabilitation. Furthermore, the training course was supervised by an ophthalmologist (N.J.M) specializing in low vision. Subjects were also provided with the Relumino manual. The manual contained detailed information about the device, its interface, and user instructions for the headset and the controller unit, as well as information on how to adjust zoom, focus, contrast, color mode, and the freeze-image option. For safety reason, patients were cautioned not to use smartphone-based LVA while in motion or walking.

Following training, subjects were given a smartphone-based LVA to use during their regular daily activities for 4 weeks. They were regularly followed-up through phone calls once per week during every week of use. The calls were made by the instructor (S.J.H.) to assist with possible troubleshooting as well as device use strategies. In addition, subjects were encouraged to use the device at school or work, as well as during daily activities where they felt comfortable using it.

### Evaluations

Visual function was assessed using three different measures, namely, visual acuity, reading performance, and facial recognition performance. For visual function measurement, free choice of visual enhancement was allowed to explore the best result achievable with a smartphone-based LVA. Subjective tests included the Korean version of the LVQOL Questionnaire. BCDVA and BCIVA were measured using a Snellen visual acuity chart at distances of 4 m and 1 m, respectively. BCNVA was measured using a Lea numbers chart at a distance of 40 cm. Visual acuities of counting fingers and hand motion were converted to the Snellen equivalent using established conversion charts^21^. All visual acuity data were converted to the logMAR before performing data analyses.

Reading performance was evaluated by measuring reading speed and accuracy using a Korean version of a reading chart based on the MNread chart design^22^. The subjects were required to read the text aloud, while the researcher recorded the completion time and errors using a stopwatch. An error was defined as any word that was skipped or mispronounced by the subject. To prevent subjects from memorizing test charts, 10 different texts of equal grammatical difficulty and word length and comparable word position were used. Reading speed was assessed by calculating the number of Korean lpm. Reading errors were not included in reading speed measurements. Reading accuracy was calculated as the ratio of the number of correctly read letters to the total number of read letters.

Face recognition performance was measured binocularly using images from the University of Pittsburgh Cohn–Kanade facial image database as previously described^12^. Subjects were asked to name the sex (male/female) and facial expressions (neutral, happy, sad, disgusted, angry, surprised, or fearful) of 50 images of faces displayed on a computer. To prevent subjects from memorizing facial images, two different image sets were used.

Vision-specific quality of life was evaluated using the validated LVQOL questionnaire. The translated version of LVQOL was administered before (Visit 1) and after the smartphone-based LVA use (Visit 3) by a researcher blinded to all clinical information. Closed-ended questions that could draw scale scored responses (from 1–5) were used, as described in previous studies^23^. The total score was calculated as the sum of the score for each item and ranged from 25 to 125. In addition, at Visit 3, subjects completed the user-feedback on their smartphone-based LVA experience (Table 4). Subjects were encouraged to answer each question as freely as possible.

### Adverse events

At regular follow-up and Visit 3, all subjects were asked whether they experienced eye strain, nausea, headache, neck pain, device site pain, or any other adverse response. In addition, to assess the effect of smartphone-based LVA on dry eye, the corneal surface was evaluated in the following order at Visits 1 and 3: standard tear film break-up time (TBUT) testing, National Eye Institute grading scheme for corneal fluorescein staining (CFS), and Schirmer I test without topical anesthesia.

### Sample size

Based on observations in a previous trial with SightPlus^14^, which reported a change in BCDVA from 0.82 ± 0.39 logMAR to 0.20 ± 0.28 logMAR, differences in the BCDVA change from baseline were statistically tested for non-inferiority. Starting from a common standard deviation (SD) of the change in BCDVA of 0.39 logMAR, and assuming an improvement from a baseline of 0.63 logMAR, a sample size of 30 patients has a 90% power of demonstrating non-inferiority, using a one-sided t-test and a significance level of 0.05.

### Statistical analysis

Data are presented as the mean ± SD where applicable. A repeated measures analysis of variance was performed to compare between visual acuity and reading performance metrics among Visits 1, 2, and 3. In case of significance, pairwise post-hoc tests with Bonferroni correction were performed. Face recognition performance and LVQOL scores were compared between Visit 1 and Visit 3 using paired Student’s t-tests. Univariate and multivariate regression analyses were performed to investigate the association between improvement of LVQOL score and clinicodemographic factors. Data were analyzed using SPSS statistical software (version 26.0, SPSS Inc., Chicago, IL, USA), and statistical significance was defined as *p* < 0.05.

## Data Availability

The datasets generated and/or analyzed during the current study are available from the corresponding author on reasonable request.
